# Supplementary material for: Hemispheric asymmetry in the human brain and in Parkinson’s disease is linked to divergent epigenetic patterns in neurons
Source: Genome Biol. 2020 Mar 9;21:61. doi: 10.1186/s13059-020-01960-1 (PMC7063821; doi:10.1186/s13059-020-01960-1)
Supplement: Supplementary file 1 — Figure S1. Isolation of human prefrontal cortex neurons by flow cytometry. Figure S2. Analysis of cytosine site coverage and sample distributions showing high technical reproducibility in discovery cohort and replication cohort. Figure S3. Hemispheric asymmetry in controls and PD patients is replicated for CpG and CpH sites with ≥100X read coverage. Figure S4. Genomic locations of differentially methylated neuronal enhancer/promoter cytosines associated with hemispheric asymmetry in controls and in PD patients. Figure S5. Genomic location of enrichment of DNA methylation changes involved in hemispheric asymmetry in human cortical neurons. Figure S6. Enhancers and promoters with DNA methylation changes in the symptom-dominant PD hemisphere have corresponding changes in the expression of their target genes. Figure S7. Hemispheric asymmetry in DNA methylation is prevalent in the human brain, as confirmed in the replication cohort. Figure S8. Epigenetic and transcriptional dysregulation is more prevalent in neurons of the symptom-dominant PD hemisphere than in neurons of the non-dominant hemisphere. Figure S9. Protein-protein interaction network of 345 proteins with altered abundance in PD relative to controls and that differed between the PD symptom-dominant and non-dominant hemisphere. Figure S10. In PD neurons, epigenetic changes with aging differ between the hemispheres matched and unmatched to symptom predominance. Figure S11. Pathways and genomic locations of DNA methylation changes relevant to hemispheric asymmetry in PD that were associated to genetic variation. Table S1. Inter-sample correlations for CpG and CpH in controls of the same hemisphere. Table S2. Software used for analysis. [file 13059_2020_1960_MOESM1_ESM.docx]

**Hemispheric asymmetry in the human brain and in Parkinson’s disease is linked to divergent epigenetic patterns in neurons**

Li P., et al.

**Additional file 1 includes:**

Figure S1-S11

Table S1-S2

**Other Supplementary Material for this manuscript includes the following:**

Additional file 2-12

**Supplementary Figures and Figure Legends**

**
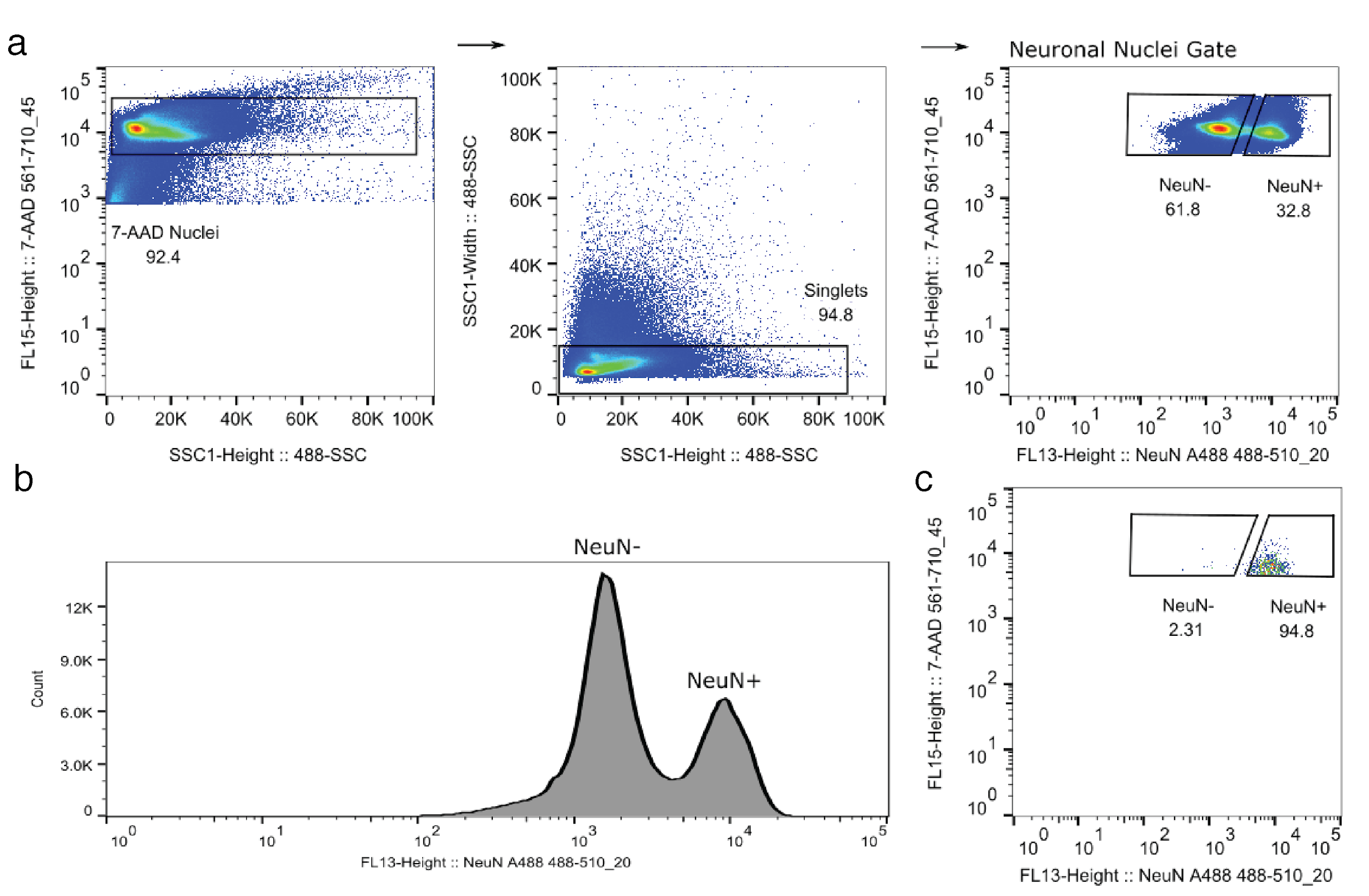
**

**Figure S1.** **Isolation of human prefrontal cortex neurons by flow cytometry.** (**a**) Representative flow-cytometry gating for the separation of single 7-AAD positive nuclei followed by sorting of neuronal nuclei (NeuN+) and glial/non-neuronal nuclei (NeuN-). (**b**) Histogram of NeuN labeled nuclei, showing two distinct populations. (**c**) Re-analysis of NeuN+ aliquots after sorting shows a high purity for NeuN+ stained nuclei (on average 94.71% ± 0.25%).

**
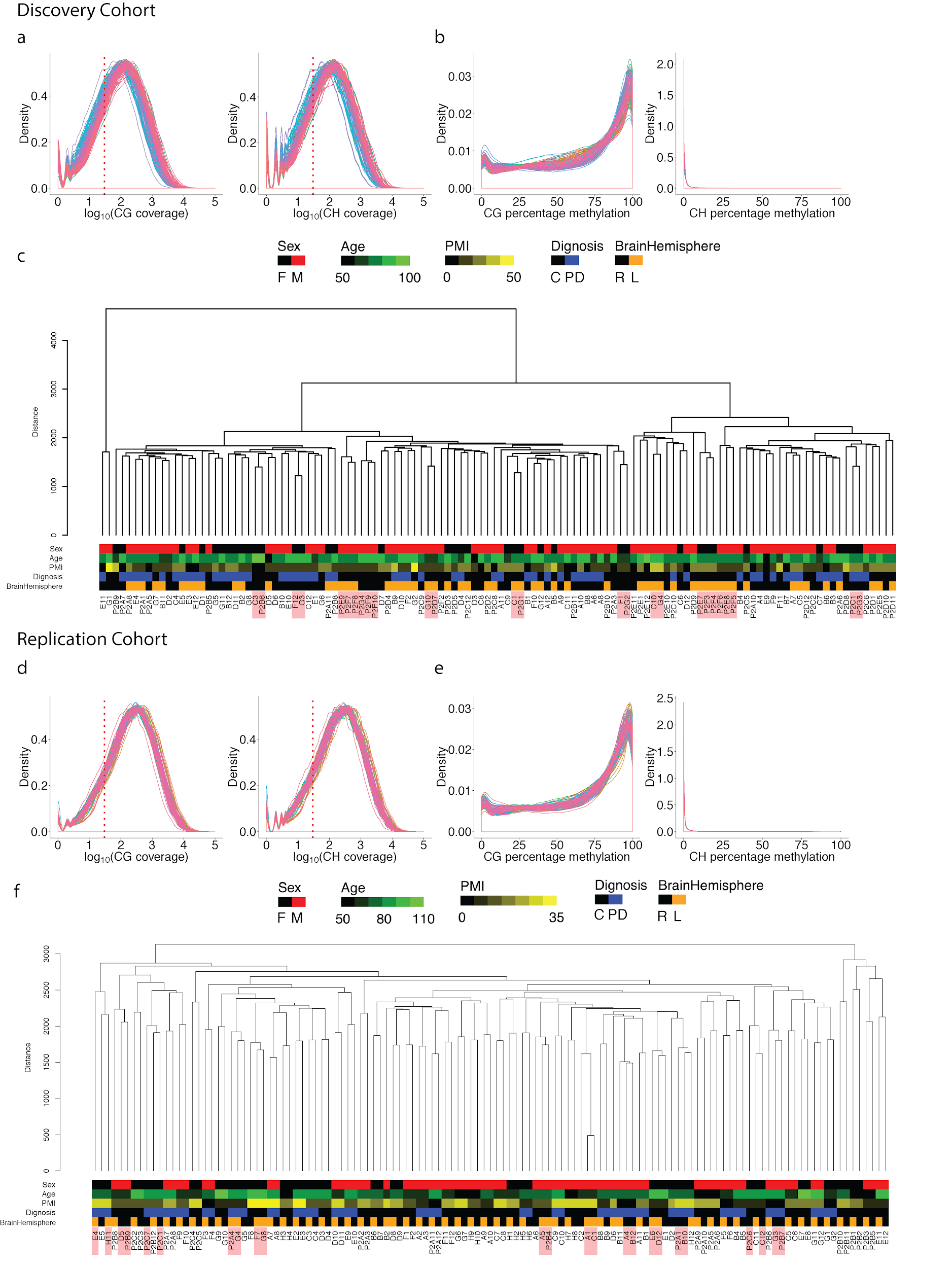
**

**Figure S2.** **Analysis of cytosine site coverage and sample distributions showing high technical reproducibility in the** **discovery cohort and replication cohort. (a, b, c)** Discovery cohort. **(d, e, f)** Replication cohort. (**a**, **d**) Density plots showing read coverage at CpG (left) and CpH (right) sites in each individual sample. The red dotted line shows 30× read coverage. (**b**, **e**) Density plots showing DNA methylation distribution at CpG (left) and CpH (right) sites having at least 30× read coverage for each sample separately. (**c**, **f**) Dendrogram showing clustering of samples based on the correlation of DNA methylation. Pearson’s correlation of DNA methylation for the 10,000 most variable CpG and CpH sites. Replicates are highlighted in red. The average correlation scores for the technical replicates were 0.94 ± 0.007 in the discovery cohort and 0.97 ± 0.003 in the replication cohort. For the replication cohort (**f**), we also observed within-sample clustering of left and right brain hemispheres for each individual.

**
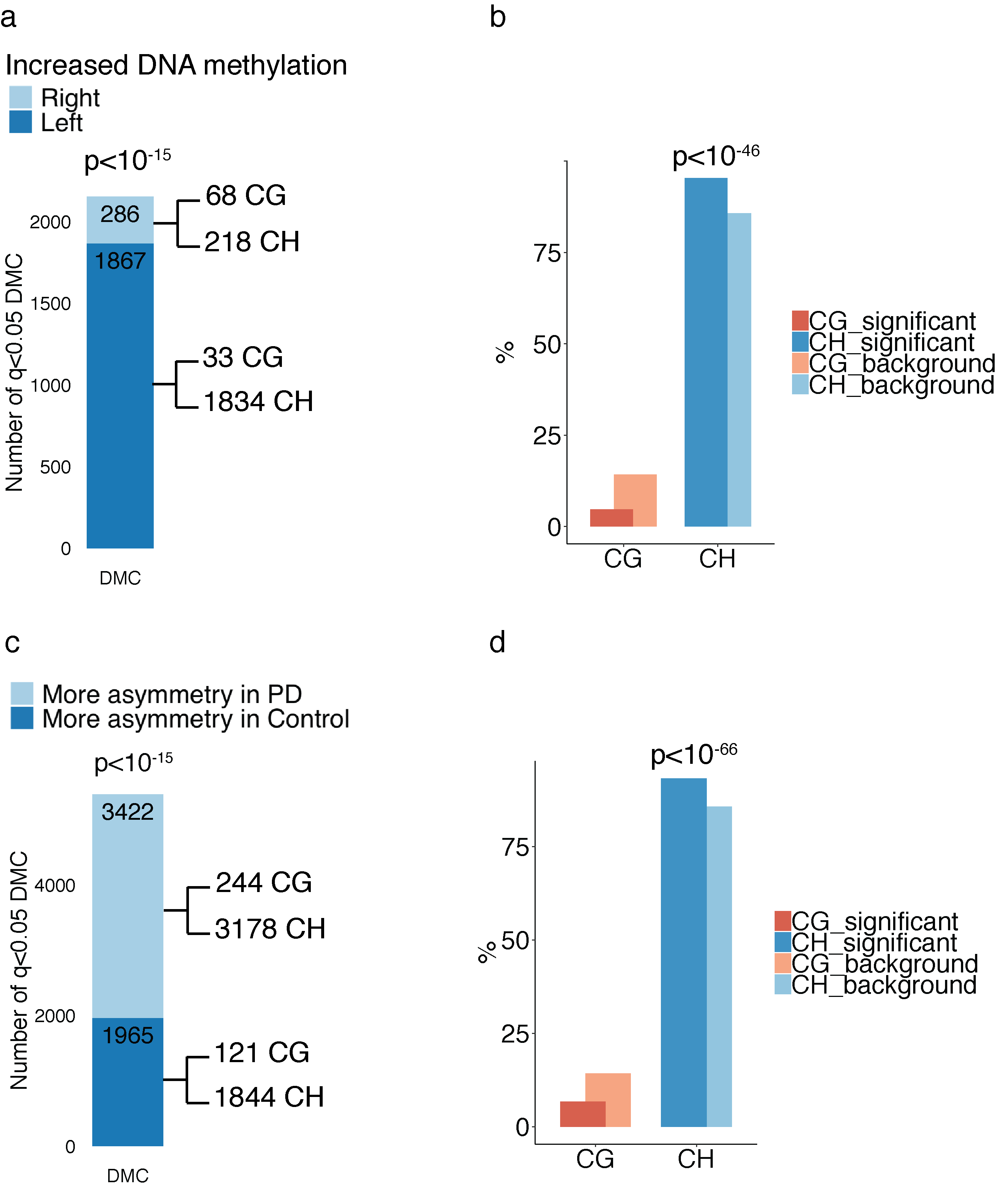
**

**Figure S3. Hemispheric asymmetry in controls and PD patients is replicated for CpG and CpH sites with ≥100X read coverage.** Analysis of 574,447 cytosine sites in the discovery cohort (n = 25 left and 23 right hemispheres of controls and 23 left and 34 right hemispheres of PD patients). **(a)** Bar plot showing the distribution of differentially methylated cytosines (DMC) across hemispheres of the control brain. The number of significant cytosine sites that show greater DNA methylation levels in the left or right hemisphere of controls is shown. The *p*-value represents the enrichment of cytosines with increased DNA methylation in the left hemisphere of controls, by Fisher's exact test. CpG and CpH site contribution to differential methylation in each hemisphere is shown. **(b)** CpG and CpH involvement in inter-hemispheric DNA methylation differences. The percent number of significantly altered CpG or CpH sites differing between hemispheres (relative to background) is shown. The *p*-value refers to the enrichment of CpH exhibiting hemispheric asymmetry, by hypergeometric test. **(c)** Comparison of the degree of hemispheric asymmetry in DNA methylation between PD patients and controls. The number of significant cytosine sites exhibiting more hemispheric asymmetry in PD or control neurons is shown. The *p*-value represents the enrichment of cytosines with greater hemispheric asymmetry in PD relative to controls, by Fisher's exact test. CpG and CpH contributions to hemispheric asymmetry are shown. **(d)** CpG and CpH involvement in hemispheric asymmetry changes in PD. The percent number of CpG or CpH sites significantly involved in hemispheric asymmetry changes in PD (relative to background) is shown. The *p*-value refers to the enrichment of CpH exhibiting hemispheric asymmetry, by hypergeometric test.

**
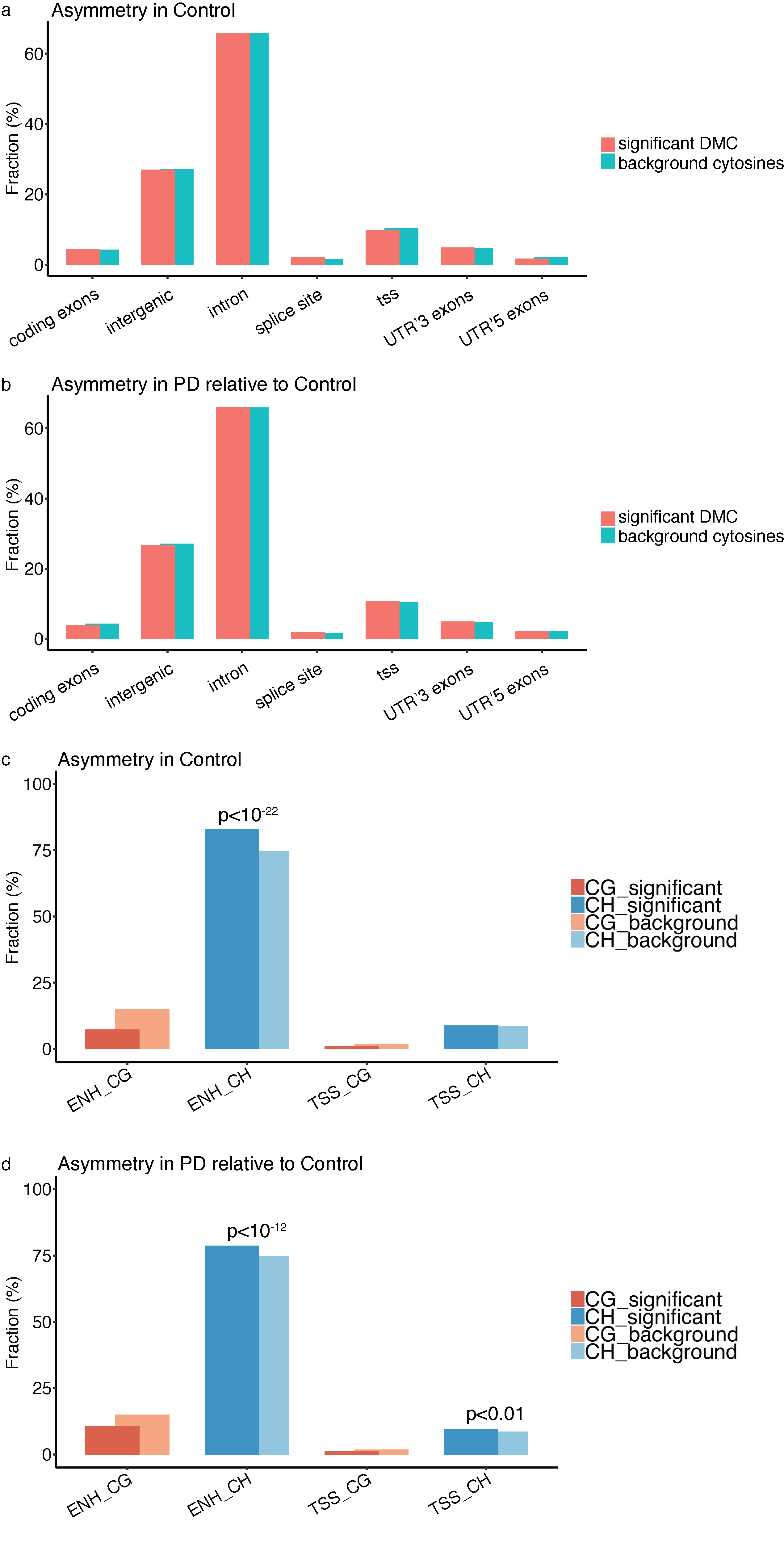
**

**Figure S4. Genomic locations of differentially methylated neuronal enhancer/promoter cytosines associated with hemispheric asymmetry in controls and in PD patients.** Location of enhancer/promoter cytosine sites relevant to hemispheric asymmetry in controls (**a, c**) and differential hemispheric asymmetry in PD patients relative to controls (**b, d**). (**a, b**) Genomic locations of enhancer/promoter cytosine sites with DNA methylation differences between hemispheres (relative to background of each location). (**c, d**) Distribution within enhancers and promoters of CpG and CpH sites exhibiting hemispheric asymmetry in DNA methylation. Enrichment of enhancers or transcription start site locations (TSS ± 2 kb) with CpG or CpH sites with differential methylation across hemispheres (CpG and CpH examined separately and relative to background). The y-axis shows the percent number of cytosines with significant hemispheric asymmetry in DNA methylation (DMC), relative to background cytosines. Enrichment determined by hypergeometric test.


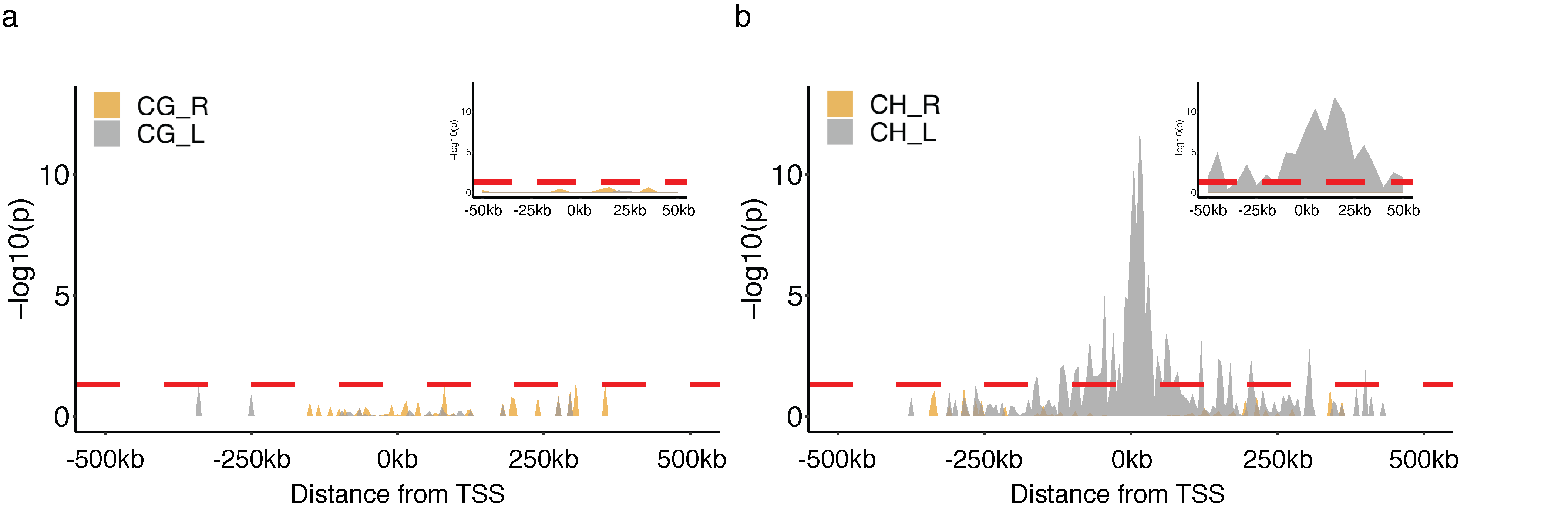


**Figure S5. Genomic location of enrichment of DNA methylation changes involved in hemispheric asymmetry in human cortical neurons.** The enrichment location of CpG (**a**) and CpH (**b**) sites with increased DNA methylation in the left or right hemisphere is shown. Location enrichment was determined by hypergeometric test of differentially methylated cytosines compared to background cytosines (5 kb bins), and relative to the distance from the nearest transcription start site. Inset is a close-up of the transcription start site region. Red dashed line, *p* < 0.05.


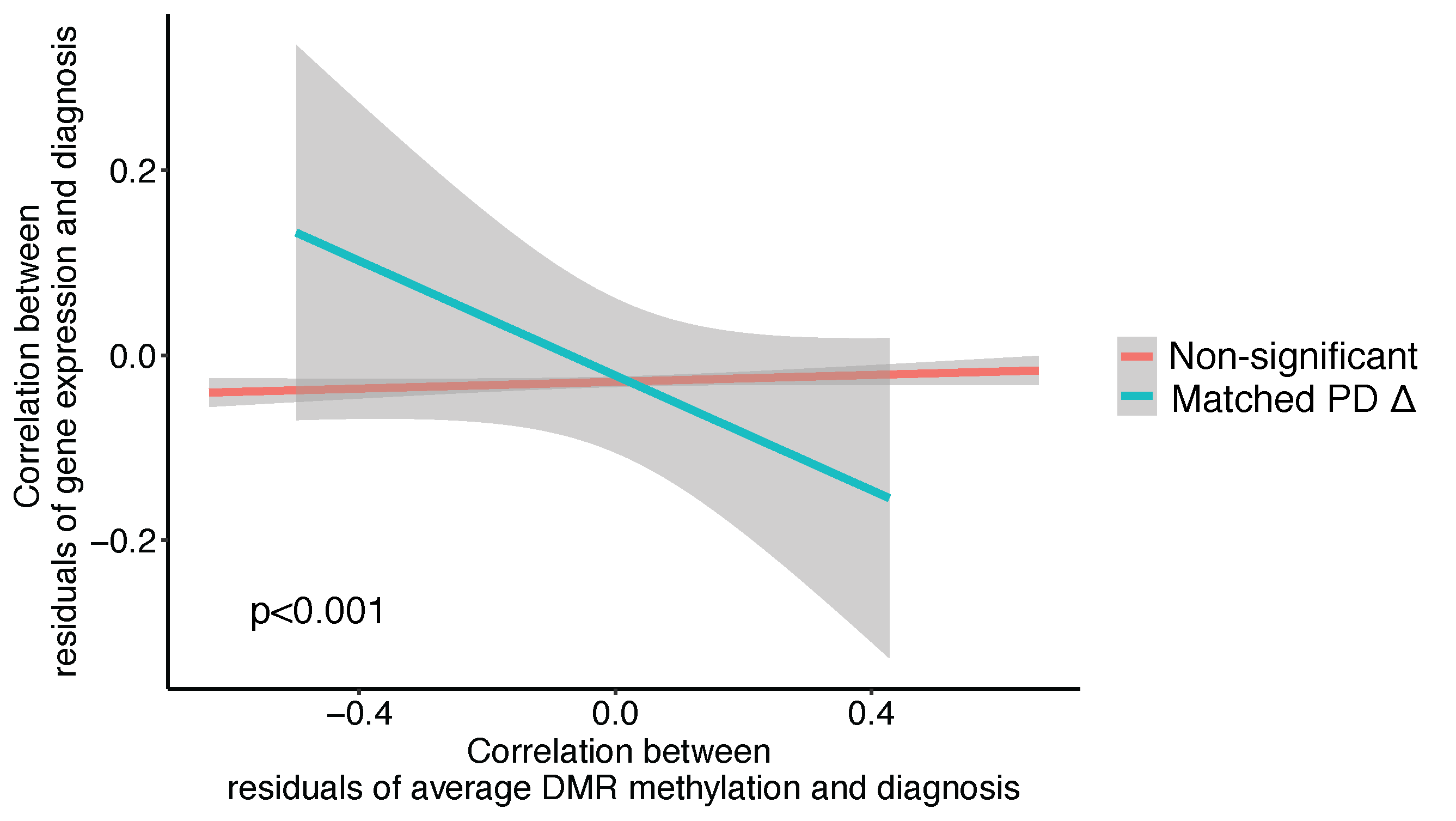


**Figure S6. Enhancers and promoters with DNA methylation changes in the symptom-dominant PD hemisphere have corresponding changes in the expression of their target genes.** RNA-seq and corresponding DNA methylation data (n = 36 individuals) was used to examine the association between gene expression status and corresponding DNA methylation status for genes with greater alterations in the PD hemisphere matched to symptom predominance. Genes with significant changes in DNA methylation at their regulatory elements exhibit an inverse correlation with their transcript level (blue line, n = 111 significant genes at *q* < 0.05). Conversely, genes with no DNA methylation changes at their regulatory elements have no associated changes in transcript level (red line, n = 6,038 non-significant genes). This signifies that DNA methylation differences at regulatory elements in PD are associated with corresponding changes in transcript levels. Correlation lines with 95% confidence interval are shown. The two correlation lines are significantly different (*p* < 0.001, by interaction term in linear regression).


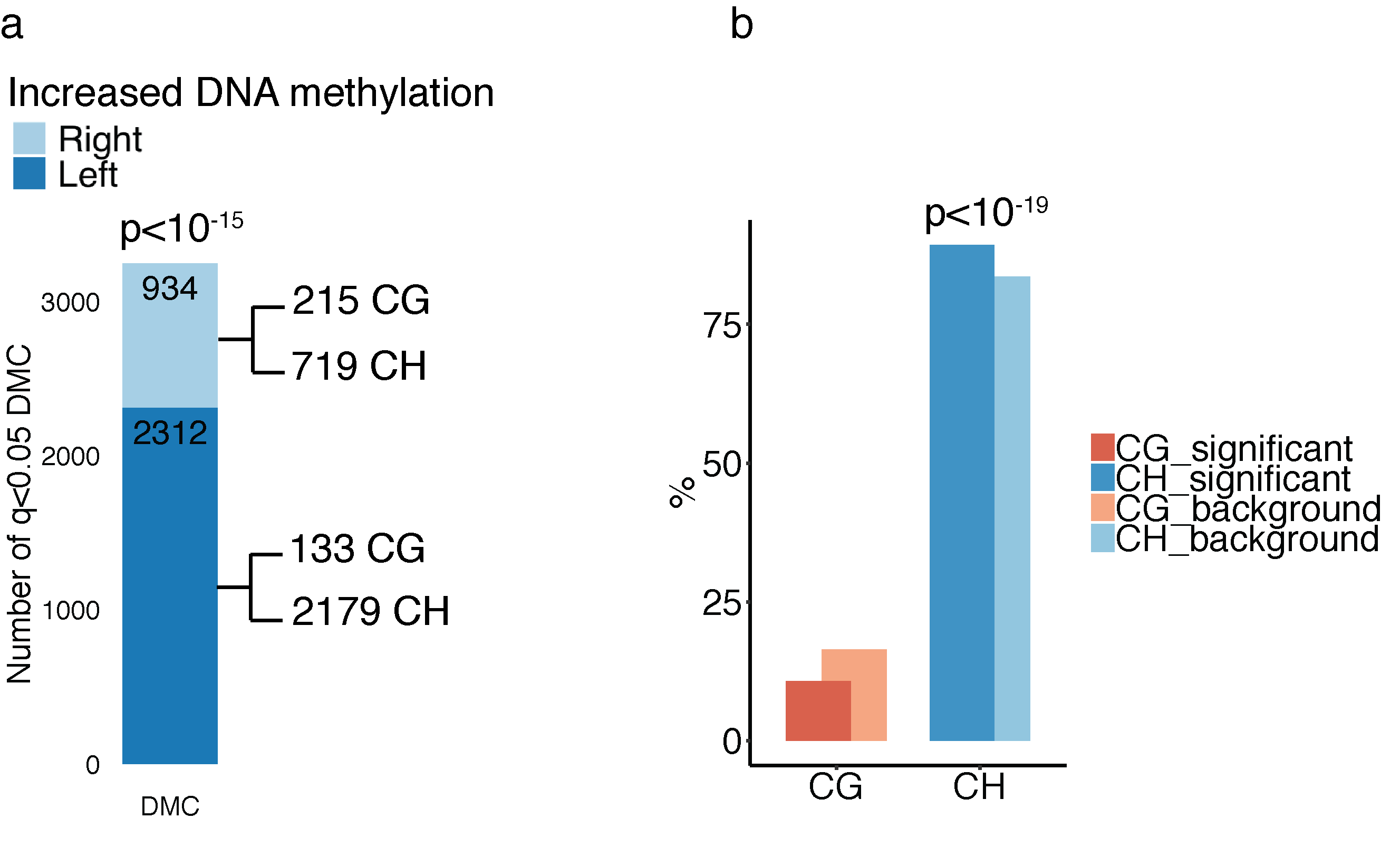


**Figure S7.** **Hemispheric asymmetry in DNA methylation is prevalent in the human brain, as confirmed in the replication cohort.** Analysis of 815,367 cytosine sites in both hemispheres of controls in the replication cohort (n = 31 controls). (**a**) Bar plot showing the distribution of differentially methylated cytosines (DMC) across hemispheres. The number of significant cytosine sites that show greater DNA methylation levels in the left or right hemisphere is shown. The *p*-value represents the enrichment of cytosines with increased DNA methylation in the left hemisphere, by Fisher's exact test. CpG and CpH site contribution to differential methylation in each hemisphere is shown. (**b**) CpG and CpH involvement in inter-hemispheric DNA methylation differences. The percent number of significantly altered CpG or CpH sites differing between hemispheres (relative to background) is shown. The *p*-value refers to the enrichment of CpH sites exhibiting hemispheric asymmetry in the human brain, by hypergeometric test.


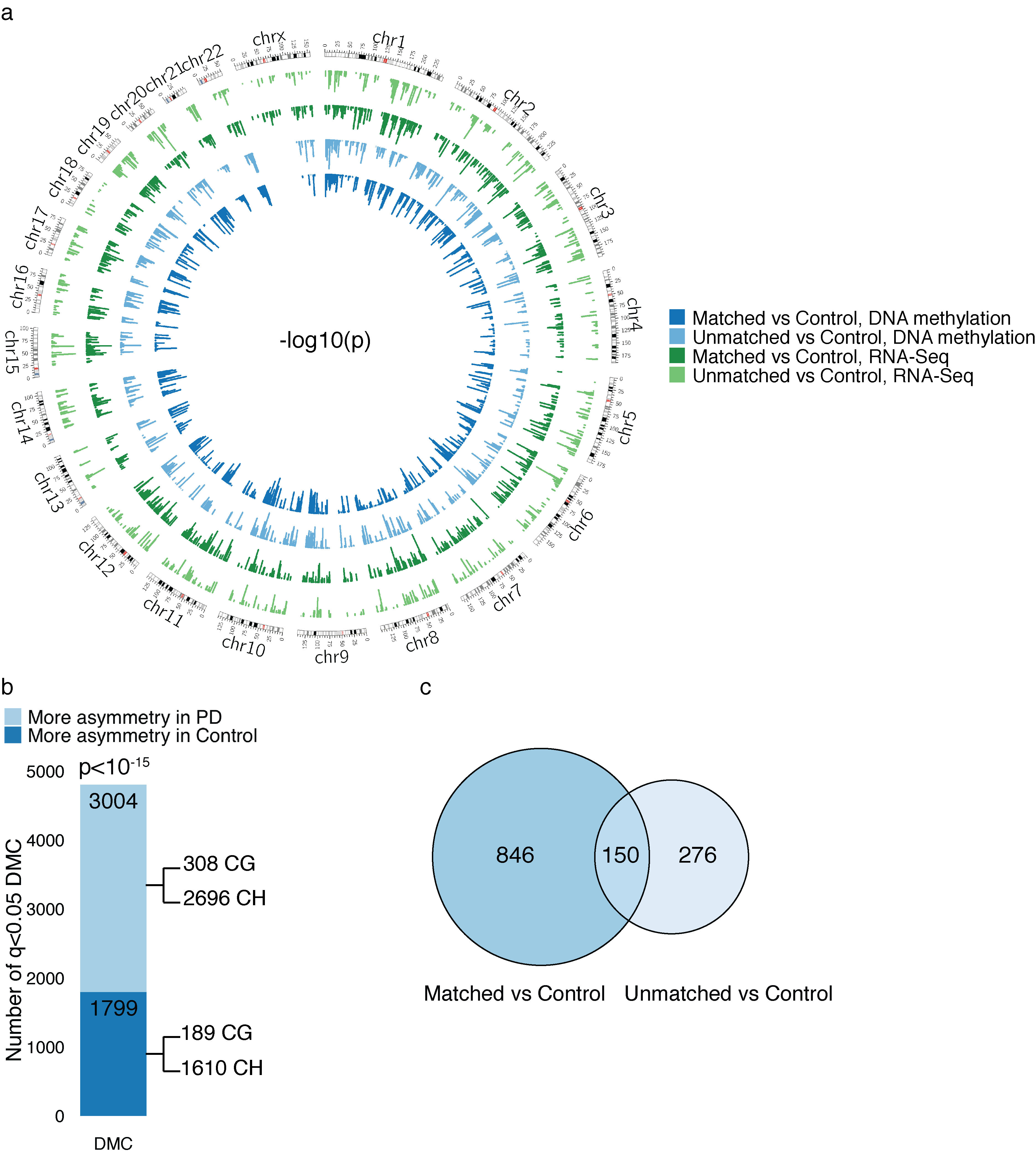


**Figure S8. Epigenetic and transcriptional dysregulation is more prevalent in neurons of the symptom-dominant PD hemisphere than in neurons of the non-dominant hemisphere.** (**a**) Circos plot showing the prevalence of DNA methylation and transcriptional changes in PD hemispheres. Differentially methylated cytosines and differentially expressed genes in neurons of the hemisphere matched or unmatched to symptom predominance relative to control neurons. Samples from the discovery DNA methylation dataset (n = 17 PD-matched, 20 PD-unmatched, 48 control hemispheres) and RNA-seq data for a subset of the same samples (n = 13 PD-matched, 11 PD-unmatched, 12 control hemispheres). -log10(p) refers to the significance of difference between PD-matched vs. control, PD-unmatched vs. control. We used a linear regression model controlling for age, sex, postmortem interval, neuronal subtype proportion, and brain hemisphere side for DNA methylation; controlling for age, sex, RIN, neuronal proportion, brain hemisphere side, and other sources of variation for RNA-seq, followed by contrasts.fit. (**b**) Comparison of the degree of hemispheric asymmetry in DNA methylation between PD patients and controls in the replication cohort (n = 26 PD and 31 controls). Significant cytosines sites exhibiting left–right hemispheric differences in DNA methylation in neurons of PD patients relative to controls were identified by robust linear regression, after controlling for age, sex, postmortem interval, and neuronal subtype proportion, followed by contrasts.fit. The number of significant cytosines sites exhibiting more hemispheric asymmetry in PD or control neurons is shown. The *p*-value represents the significance of greater hemispheric asymmetry in PD (*p* < 10^-15^, Fisher's exact test). CpG and CpH contributions to hemispheric asymmetry are shown. (**c**) DNA methylation differences in the replication cohort that are relevant to the lateralization of PD symptoms. Venn diagram showing the number of genes with epigenetic abnormalities in the PD hemisphere matched or unmatched to symptom predominance, relative to control hemispheres (replication cohort, n = 24 PD-matched, 26 PD-unmatched, 61 control hemispheres).

**

**

**Figure S9. Protein-protein interaction network of 345 proteins with altered abundance in PD relative to controls and that differed between the PD symptom-dominant and non-dominant hemisphere.** Proteins differing between PD patients and controls (n = 3 per diagnosis group) and between the symptom-dominant and non-dominant hemispheres (n = 5 PD patients, both hemispheres) were identified. Network analysis performed by STRING. Proteins of genes linked to PD risk (familial and/or identified by GWAS [50]) are highlighted in yellow. Red indicates upregulation and blue indicates downregulation of proteins in the symptom-dominant hemisphere of PD patients. NCAM1 is highlighted by a black box.

**
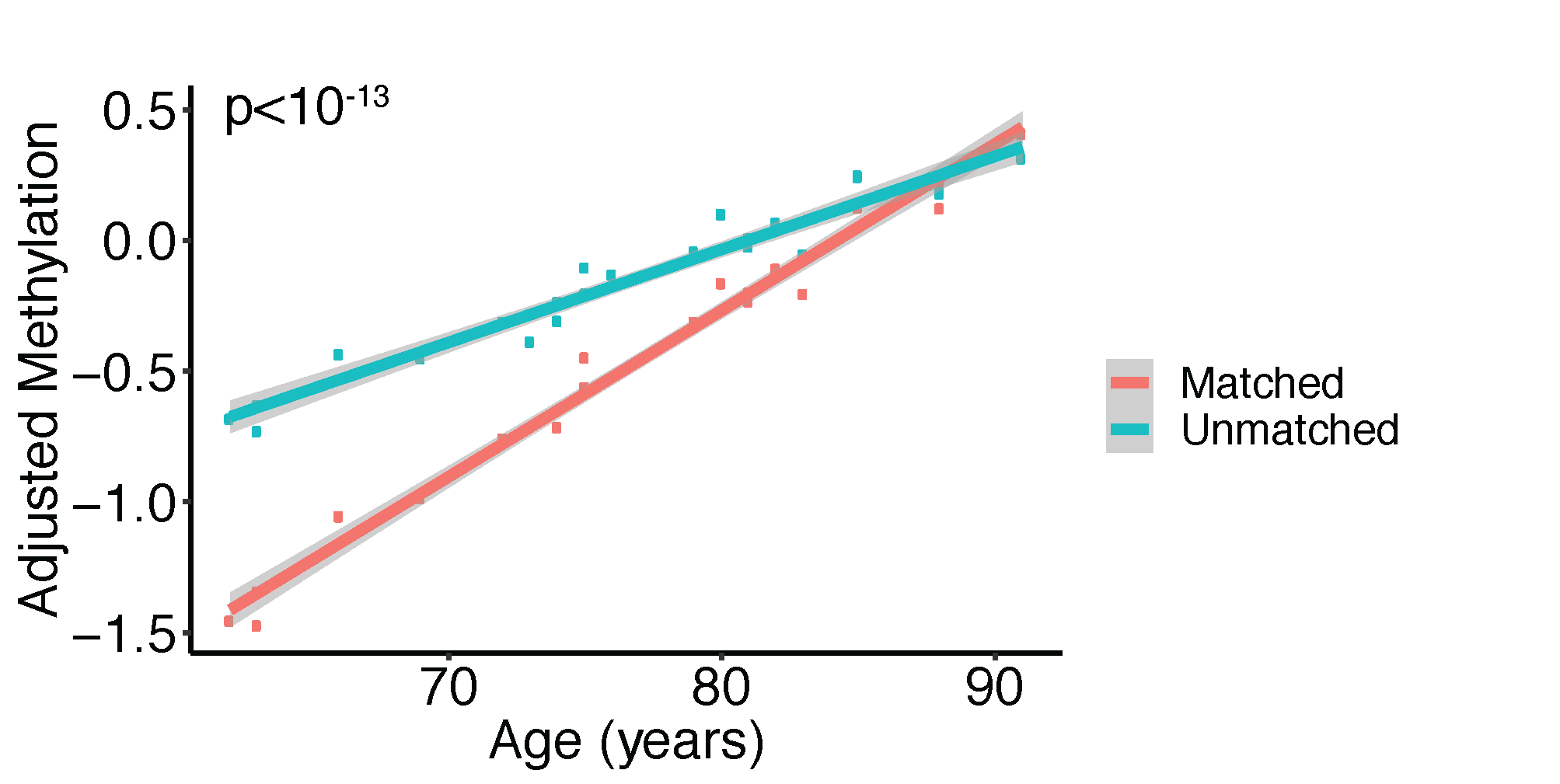
**

**Figure S10. In PD neurons, epigenetic changes with aging differ between the hemispheres matched and unmatched to symptom predominance (n = 24 PD-matched, 26 PD-unmatched).** Scatter plot showing adjusted DNA methylation changes with age in the PD hemisphere matched or unmatched to symptom predominance. The grey area represents confidence intervals. Differential aging between the symptom-dominant and non-dominant hemispheres occurred at 4,568 DNA methylation sites at enhancers and promoters, after adjusting for sex, postmortem interval, brain hemisphere (left or right), and neuronal subtype proportion (*q* < 0.05, robust linear regression with contrasts). Aging changes in DNA methylation were greater in the hemisphere matched to PD symptom predominance compared to the unmatched hemisphere (*p* < 10^-13^, linear regression with interaction term). The convergence of neuronal DNA methylation between the symptom-dominant and non-dominant hemisphere with aging may underlie the typical gradual transition from asymmetric to bilateral symptom presentation in PD [11].

**
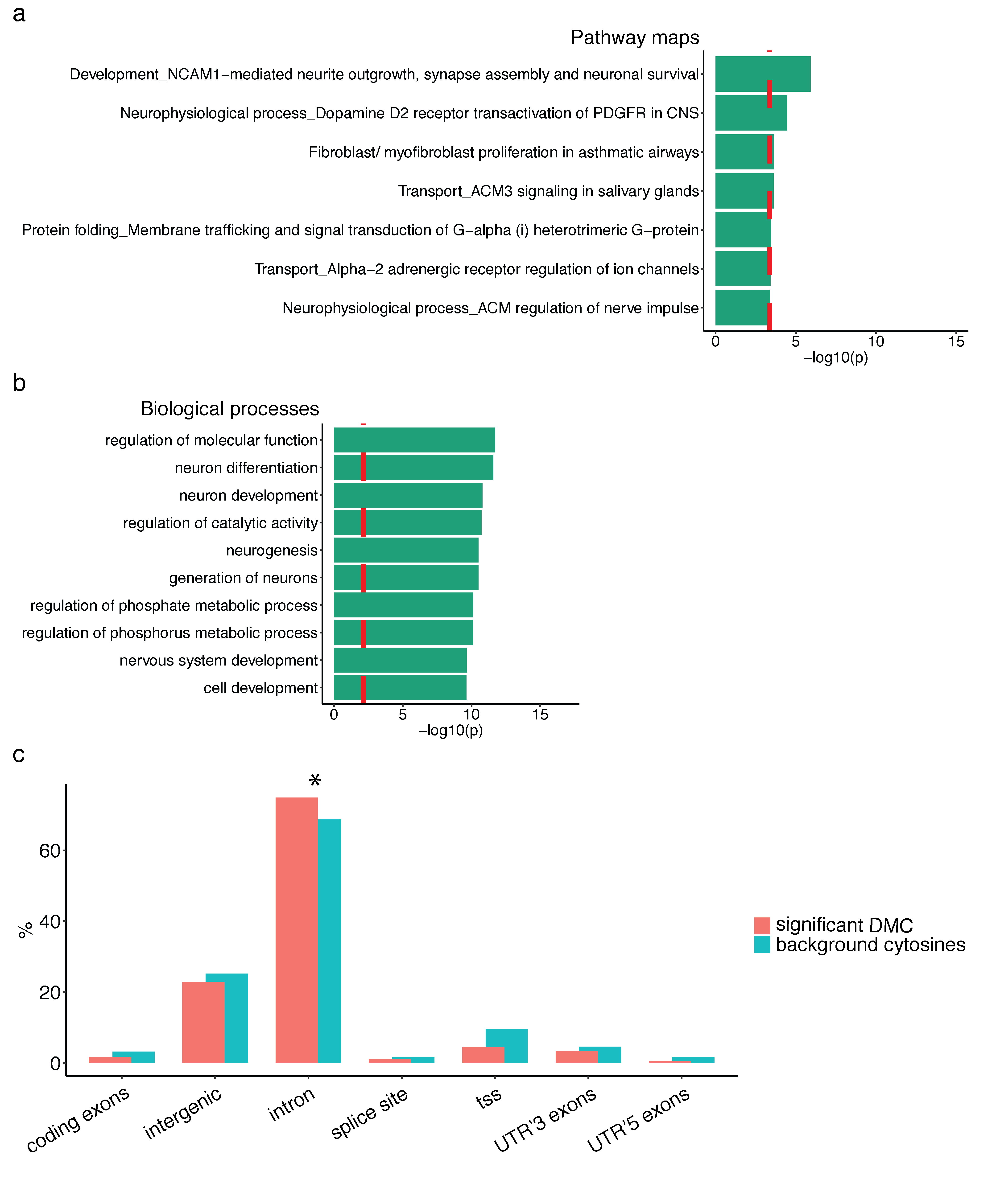
**

**Figure S11. Pathways and genomic locations of DNA methylation changes relevant to hemispheric asymmetry in PD that were associated to genetic variation.** meQTL analysis examining the effects of genotype on DNA methylation, adjusting for diagnosis, hemisphere, age, sex, postmortem interval, and neuronal subtypes (replication cohort, left and right hemispheres of n = 31 controls and 26 PD patients). Pathway maps (**a**) and biological processes (**b**) of SNP-associated DNA methylation sites relevant to hemispheric asymmetry in PD. Pathway analysis performed using MetaCore and *q* < 0.05 pathways are shown. (**c**) Genomic location of DNA methylation sites with inter-hemispheric differences in PD that are associated with genetic variation. Y-axis shows the percent number of SNP-associated DNA methylation sites relevant to hemispheric asymmetry in PD, relative to background cytosines. **p <* 0.05, hypergeometric test.

**Table S1.** Inter-sample correlations for CpG and CpH in controls of the same hemisphere.

|  | CG/CH | Hemisphere | 1st Qu. | Median | 3rd Qu. |
| --- | --- | --- | --- | --- | --- |
| Top 10K^†^ | CG | L | 0.536 | 0.610 | 0.682 |
|  |  | R | 0.488 | 0.619 | 0.689 |
|  | CH | L | 0.589 | 0.637 | 0.674 |
|  |  | R | 0.585 | 0.630 | 0.662 |
| All | CG | L | 0.910 | 0.926 | 0.937 |
|  |  | R | 0.901 | 0.924 | 0.937 |
|  | CH | L | 0.839 | 0.862 | 0.883 |
|  |  | R | 0.837 | 0.858 | 0.875 |

^†^Top 10,000 most variable cytosine sites (CpG and CpH sites with the highest standard deviation across samples)

**Table S2.** Software used for analysis

| Name | Version | URL |
| --- | --- | --- |
| Tissue Specific Enhancers |  | https://epigenome.wustl.edu/TSE/browse.php |
| ppDesigner | v2.0 | http://genome-tech.ucsd.edu/public/Gen2_BSPP/ppDesigner/ppDesigner.php |
| 1000 Genomes Project |  | http://www.internationalgenome.org/ |
| Trimmomatic | v0.32 | http://www.usadellab.org/cms/index.php?page=trimmomatic |
| Bismark | v0.17.0 | https://www.bioinformatics.babraham.ac.uk/projects/bismark/ |
| CIBERSORT |  | http://cibersort.stanford.edu |
| R | v3.5.1 | https://www.r-project.org/ |
| limma | v3.30.13 | https://bioconductor.org/packages/release/bioc/html/limma.html |
| lumi | v2.30.0 | https://www.bioconductor.org/packages/release/bioc/html/lumi.html |
| TrimGalore | v0.4.4 | https://www.bioinformatics.babraham.ac.uk/projects/trim_galore/ |
| HiCUP | v0.5.9 | https://www.bioinformatics.babraham.ac.uk/projects/hicup/ |
| HOMER | v4.9.1 | http://homer.ucsd.edu/homer/interactions2/ |
| GREAT | v4.0.4 | http://great.stanford.edu/public/html/ |
| STAR | v2.3.5a | https://code.google.com/archive/p/rna-star/ |
| edgeR | v3.16.5 | https://bioconductor.org/packages/release/bioc/html/edgeR.html |
| RUVseq | v1.18.0 | https://bioconductor.org/packages/release/bioc/html/RUVSeq.html |
| stats | v3.3.3 | https://stat.ethz.ch/R-manual/R-devel/library/stats/html/00Index.html |
| MetaCore |  | https://clarivate.com/products/metacore/ |
| g:Profiler |  | https://biit.cs.ut.ee/gprofiler/gost |
| STRING | v11.0 | https://string-db.org/ |
| GSEA | v3.0 | http://software.broadinstitute.org/gsea/index.jsp |
| Cytoscape | v3.7.1 | http://cytoscape.org/ |
| RobustRankAggreg | v1.1 | https://cran.r-project.org/web/packages/RobustRankAggreg/index.html |
