# Supplementary material for: Hemispheric asymmetry in the human brain and in Parkinson’s disease is linked to divergent epigenetic patterns in neurons
Source: Genome Biol. 2020 Mar 9;21:61. doi: 10.1186/s13059-020-01960-1 (PMC7063821; doi:10.1186/s13059-020-01960-1)
Supplement: Supplementary file 12 — Review history. [file 13059_2020_1960_MOESM12_ESM.docx]

Review History

**First round of review**

**Reviewer 1**

**Are you able to assess all statistics in the manuscript, including the appropriateness of statistical tests used?**

Yes, and I have assessed the statistics in my report.

**Comments to author:**

This is a potentially interesting and important study examining DNA methylation of known cortical regulatory domains (enhancers, promoters) in the right-left lateralization of human brain in controls and differences in Parkinson disease brain. The authors demonstrate that methylation of CH, not CpG, sites is responsible for hemispheric differences in controls, at regulatory regions for genes involved in neronal development. They further show that PD brains are characterized by a greater hemispheric asymmetry, also due to CH methylation, but over genes involved in immune responses and synapses and near SNPs with known genetic risk for PD. Lastly, they show that PD brains with a longer disease course show greater methylation asymmetry, and that hemispheric asymmetry is gradually lost with aging in controls. Overall, this is a potentially important addition to the literature, however, there are several concerns about the methods and conclusions that would need to be addressed.

1. There are major concerns about the padlock probe approach and whether this method is capable of quantifying CH methylation at single base resolution. This is because CH methylation of any individual site is <1-2%, so most other approaches use larger windows to capture CH methylation. The authors need to assess the single base correlation of CpH compared to CpG methylation among individual controls of the same hemisphere. Presumably it will be much lower inter individual correlation for CpH compared to CpG, which raises concerns about the authors' main conclusion about CH methylation differences between hemispheres being a biologically relevant finding. If the results in Figures 1 and 2 are correct in showing large CH methylation differences between L and R hemispheres, and in PD vs controls, this should also be detectable by another method. The authors need to demonstrate by low coverage WGBS with 20 kb windows (or another comparable method) that there are detectable significant differences in %CH methylation between hemispheres. Without confirmation by another method, there remains a major concern that the presumed CH methylation differences could be due to technical artifacts.
2. The abstract is quite vague. It is really impossible to know from reading it what approaches were taken and the results are all more like conclusions rather than stating quantitatively what results were observed.
3. There are several places in the manuscript where the conclusions about DNA methylation differences detected are overstated. First, in the title, "Epigenetic contributions to" should be replaced with something more like "Epigenetic differences in". The last sentence of the Introduction reading "Together, our results support that hemispheric asymmetry in PD involves the disrupted epigenetic regulation of genes, which may enable asymmetric neuronal dysfunction and the contralateral predominance of PD symptoms" is not supported by the data and should be similarly reworded to remove the implication of epigenetic causality. Also, in the Discussion, statements such as "CpH silencing of gene activity" are not appropriate because neither this or prior studies of CH methylation have established its causality in gene repression.
4. The Discussion did not really address the main findings of the manuscript and how they fit into the existing literature on the DNA methylation landscape of the brain in health and disease. CpH methylation in development and other disorders should be discussed, particularly. It is still unclear if CpH methylation in neurons is a cause or consequence of differential gene expression patterns.

**Reviewer 2**

**Are you able to assess all statistics in the manuscript, including the appropriateness of statistical tests used?**

No, I do not feel adequately qualified to assess the statistics.

**Comments to author:**

In this manuscript, Li et al, investigated how DNA methylation correlates with the hemispheric asymmetry phenotype in healthy and PD neurons. This is an interesting biological question, and I like the authors made an effort to gather a large number of individuals for this study. They profiled DNA methylation of selective regulatory regions defined by the Epigenome Roadmap project and showed differential methylation between the neurons from the left and right hemispheres. The author further validated the results by an independent set of cohort. The experimental design is sounds, but the manuscript falls short on analysis.

1. The authors show that the majority of the methylation differences are from CH methylation in normal brain samples. Did the author separate the individuals with left or right preference? I assume the methylation will correlate with this preference, but I can't figure out through the manuscript whether this has been corrected before the analysis.

2. When analysis these sites, they should separate the CpG and CH sites into three different groups: promoter, distal regulatory regions, and gene body. Depends on the genomic location, CpG methylation is correlated with various genomic activities.

3. Figure 2c, where shows the genomic distances to TSS for differential methylated sites, the y-axis should be scaled at the same level between the left and right plot. The current plots are too zoomed out, therefore hard to tell the distance from TSS, which was only average at 47kb, while the intervals on the plots are at 250kb. To better display the data, I suggest the authors use a CDF plot to show the distribution of distance to TSS.

4. The authors used Schmitt et al., 2017 Cell Reports to link distal regions to target genes. However, HiC datasets were not sequenced deep enough to call interactions between gene and regulatory regions reliably. The authors should use Song et al., Nature Genetics 2019 in which they mapped promoter-centric interactions genome-wide in excitatory neurons and Jung et al., Nature Genetics 2019 with promoter centric interactions data in brain tissues. This analysis affects figure 1e, 2a, 2d, 2e,3c, and 3d.

5. Similarly, in Figure 2a, where the authors tried to show PD related genes show differential methylation, it was not clear whether this is based on TSS or distal region methylation (either CG or CH). How do these methylation differences affect gene expression? Is this PD gene more enriched than the background?

6. I had a tough time understanding Figures 2d and 2e. I know the authors were trying to convey the idea of in PD samples specific methylation difference, but I suggest the authors make the figures more readable to the audience. Figure 1e has too many dimensions and doesn't need to be this complicated to show the same message. On the other hand, the corresponding text for explaining the results was too concise, offering little biological insights. This makes me feel they performed this analysis to fill in the figure without providing enough biological insight. This is when a lot of readers start to lose interest in the manuscript.

7. I like the idea of replicating the study with an independent cohort. This is well done. But again, I would like to suggest changing the analysis of assigning target genes to distal regions with higher resolution HiC datasets. Also, consider an individual's preference of the left or right side in both control and PD samples when possible, as such information probably will help to provide cleaner analysis results.

8. The authors next perform proteomic analysis to identified differential proteins in PD samples. Is this protein level analysis correlated with the RNA-seq and methylome analysis?

9. The section of "changes in hemispheric asymmetry in neurons with aging" is not well presented. Figure 4a needs labeling # of rows, and what are the different time points used for aging? Figure 4b, how are the values for each dot calculated? Is this a genome-wide average?

10. the last part of me-QTL analysis is interesting; besides some description, there is no figure or table associated with these results. What are the biological contributions of me-QTL to PD? What are the genes associated with me-QTL?

**Authors Response**

**Point-by-point responses to the reviewers’ comments:**

Reviewer #1

Comment 1: There are major concerns about the padlock probe approach and whether this method is capable of quantifying CH methylation at single base resolution. This is because CH methylation of any individual site is <1-2%, so most other approaches use larger windows to capture CH methylation. The authors need to assess the single base correlation of CpH compared to CpG methylation among individual controls of the same hemisphere. Presumably it will be much lower inter individual correlation for CpH compared to CpG, which raises concerns about the authors' main conclusion about CH methylation differences between hemispheres being a biologically relevant finding. If the results in Figures 1 and 2 are correct in showing large CH methylation differences between L and R hemispheres, and in PD vs controls, this should also be detectable by another method. The authors need to demonstrate by low coverage WGBS with 20 kb windows (or another comparable method) that there are detectable significant differences in %CH methylation between hemispheres. Without confirmation by another method, there remains a major concern that the presumed CH methylation differences could be due to technical artifacts.

*Response 1: We thank the reviewer for their interest in our paper and helpful suggestions. In this study we profile DNA methylation at enhancers and promoters using a deep-sequencing bisulfite approach known as bisulfite padlock probes. This approach profiles CpG and CpH sites at single base resolution, and has high read coverage at each site (in contrast to WGBS). Further analysis showed similar CpG and CpH inter-sample correlations for controls of the same hemisphere. Also, we reproduced our findings for hemispheric asymmetry in DNA methylation in controls and in Parkinson’s disease (PD) patients in an analysis restricted to CpG and CpH sites reaching ≥100X read coverage. We also reproduced our findings on hemispheric asymmetry in the human brain using the replication cohort. Together, these analyses support the robustness of our findings.

Additional details: In response to the reviewer comments, we performed a single base correlative analyses to compare CpG and CpH sites in controls of the same hemisphere. The inter-sample correlations for CpG and CpH are similar; both for the top 10,000 most variable cytosine sites (10K CpG and 10K CpH sites with the highest standard deviation across samples) and for all the CpG and CpH probes assayed. This is presented in the new Supplementary Table 1 (shown below). We also have added the following text to the Results:

Results, pg 6. Also, inter-sample correlations for CpG and CpH were similar (Table S1).
 
Table S1. Inter-sample correlations for CpG and CpH in controls of the same hemisphere.
CG/CH Hemisphere 1st Qu. Median 3rd Qu.
Top 10K
CG L 0.536 0.610 0.682
R 0.488 0.619 0.689
CH L 0.589 0.637 0.674
R 0.585 0.630 0.662
All CG L 0.910 0.926 0.937
R 0.901 0.924 0.937
CH L 0.839 0.862 0.883
R 0.837 0.858 0.875

The bisulfite padlock probe approach is a deep-sequencing approach, allowing us to profile CpG and CpH sites with single base resolution. In our many prior studies with this technique, we have determined that DNA methylation findings are consistent with a ≥30× read depth per cytosine site (CpG and CpH) [1-5]. In comparison, WGBS involves low read coverage and a window-based analysis of sites across numerous kilobases to overcome single nucleotide reliability concerns, which could result in an inconclusive attempt to confirm our deep-sequencing approach. However, in response to the reviewer comment we examined hemispheric asymmetry in controls and PD patients for CpG and CpH sites with 100× read coverage. We again found that neurons in the left hemisphere of controls had higher DNA methylation levels than in the right hemisphere (p<10-15, Fisher's exact test), with CpH methylation being the predominant contributor to hemispheric asymmetry in DNA methylation (p<10-46 for CpH enrichment, hypergeometric test). PD patients exhibited more hemispheric asymmetry in DNA methylation than controls (p<10-15, Fisher’s exact test). CpH sites were again the primary contributor to hemispheric asymmetry in neuronal epigenomes of PD patients (p<10-66 for CpH enrichment, hypergeometric test). These analyses are presented in the new Figure S3.

We also validated our finding that hemispheric asymmetry in DNA methylation is prevalent in the human brain using the controls in the replication cohort. We replicated that neurons in the left hemisphere of controls had higher DNA methylation levels than in the right hemisphere (p<10-15, Fisher's exact test), with CpH methylation being the predominant contributor to hemispheric asymmetry in DNA methylation (p<10-19 for CpH enrichment, hypergeometric test). These analyses are presented in the new Figure S7.

Figure S3. Hemispheric asymmetry in controls and PD patients is replicated for CpG and CpH sites with ≥100X read coverage. Analysis of 574,447 cytosine sites in the discovery cohort (n = 25 left and 23 right hemispheres of controls and 23 left and 34 right hemispheres of PD patients). (a) Bar plot showing the distribution of differentially methylated cytosines (DMC) across hemispheres of the control brain. The number of significant cytosine sites that show greater DNA methylation levels in the left or right hemisphere of controls is shown. The p-value represents the enrichment of cytosines with increased DNA methylation in the left hemisphere of controls, by Fisher's exact test. CpG and CpH site contribution to differential methylation in each hemisphere is shown. (b) CpG and CpH involvement in inter-hemispheric DNA methylation differences. The percent number of significantly altered CpG or CpH sites differing between hemispheres (relative to background) is shown. The p-value refers to the enrichment of CpH exhibiting hemispheric asymmetry, by hypergeometric test. (c) Comparison of the degree of hemispheric asymmetry in DNA methylation between PD patients and controls. The number of significant cytosine sites exhibiting more hemispheric asymmetry in PD or control neurons is shown. The p-value represents the enrichment of cytosines with greater hemispheric asymmetry in PD relative to controls, by Fisher's exact test. CpG and CpH contributions to hemispheric asymmetry are shown. (d) CpG and CpH involvement in hemispheric asymmetry changes in PD. The percent number of CpG or CpH sites significantly involved in hemispheric asymmetry changes in PD (relative to background) is shown. The p-value refers to the enrichment of CpH exhibiting hemispheric asymmetry, by hypergeometric test.

Figure S7. Hemispheric asymmetry in DNA methylation is prevalent in the human brain, as confirmed in the replication cohort. Analysis of 815,367 cytosine sites in both hemispheres of controls in the replication cohort (n = 31 controls). (a) Bar plot showing the distribution of differentially methylated cytosines (DMC) across hemispheres. The number of significant cytosine sites that show greater DNA methylation levels in the left or right hemisphere is shown. The p-value represents the enrichment of cytosines with increased DNA methylation in the left hemisphere, by Fisher's exact test. CpG and CpH site contribution to differential methylation in each hemisphere is shown. (b) CpG and CpH involvement in inter-hemispheric DNA methylation differences. The percent number of significantly altered CpG or CpH sites differing between hemispheres (relative to background) is shown. The p-value refers to the enrichment of CpH sites exhibiting hemispheric asymmetry in the human brain, by hypergeometric test.

References
1: Li P, Marshall L, Oh G, Jakubowski JL, Groot D, He Y, Wang T, Petronis A, Labrie V. Epigenetic dysregulation of enhancers in neurons is associated with Alzheimer's disease pathology and cognitive symptoms. Nat Commun. 2019 May 21;10(1):2246. doi: 10.1038/s41467-019-10101-7. PubMed PMID: 31113950; PubMed Central PMCID: PMC6529540.

2: Oh G, Koncevičius K, Ebrahimi S, Carlucci M, Groot DE, Nair A, Zhang A, Kriščiūnas A, Oh ES, Labrie V, Wong AHC, Gordevičius J, Jia P, Susic M, Petronis A. Circadian oscillations of cytosine modification in humans contribute to epigenetic variability, aging, and complex disease. Genome Biol. 2019 Jan 3;20(1):2. doi: 10.1186/s13059-018-1608-9. PubMed PMID: 30606238; PubMed Central PMCID: PMC6317262.

3: Oh G, Ebrahimi S, Carlucci M, Zhang A, Nair A, Groot DE, Labrie V, Jia P, Oh ES, Jeremian RH, Susic M, Shrestha TC, Ralph MR, Gordevičius J, Koncevičius K, Petronis A. Cytosine modifications exhibit circadian oscillations that are involved in epigenetic diversity and aging. Nat Commun. 2018 Feb 13;9(1):644. doi: 10.1038/s41467-018-03073-7. PubMed PMID: 29440637; PubMed Central PMCID: PMC5811577.

4: Oh E, Jeremian R, Oh G, Groot D, Susic M, Lee K, Foy K, Laird PW, Petronis A, Labrie V. Transcriptional heterogeneity in the lactase gene within cell-type is linked to the epigenome. Sci Rep. 2017 Jan 31;7:41843. doi: 10.1038/srep41843. PubMed PMID: 28139744; PubMed Central PMCID: PMC5282553.

5: Labrie V, Buske OJ, Oh E, Jeremian R, Ptak C, Gasiūnas G, Maleckas A, Petereit R, Žvirbliene A, Adamonis K, Kriukienė E, Koncevičius K, Gordevičius J, Nair A, Zhang A, Ebrahimi S, Oh G, Šikšnys V, Kupčinskas L, Brudno M, Petronis A. Lactase nonpersistence is directed by DNA-variation-dependent epigenetic aging. Nat Struct Mol Biol. 2016 Jun;23(6):566-73. doi: 10.1038/nsmb.3227. Epub 2016 May 9. PubMed PMID: 27159559; PubMed Central PMCID: PMC4899171.*

________________________________________

Comment 2: The abstract is quite vague. It is really impossible to know from reading it what approaches were taken and the results are all more like conclusions rather than stating quantitatively what results were observed.

*Response 2: We have revised the abstract to include more information on the approaches taken and the results observed. The abstract now states:

Background
Hemispheric asymmetry in neuronal processes is a fundamental feature of the human brain and drives symptom lateralization in Parkinson's disease (PD), but its molecular determinants are unknown. Here, we identify divergent epigenetic patterns involved in hemispheric asymmetry by profiling DNA methylation in isolated prefrontal cortex neurons from control and PD brain hemispheres. DNA methylation is fine-mapped at enhancers and promoters, genome-wide, by targeted bisulfite sequencing in two independent sample cohorts.

Results
We find that neurons of the human prefrontal cortex exhibit hemispheric differences in DNA methylation. Hemispheric asymmetry in neuronal DNA methylation patterns is largely mediated by differential CpH methylation, and chromatin conformation analysis finds that it targets thousands of genes. With aging, there is a loss of hemispheric asymmetry in neuronal epigenomes, such that hemispheres epigenetically converge in late life. In neurons of PD patients, hemispheric asymmetry in DNA methylation is greater than in controls and involves many PD risk genes. Epigenetic, transcriptomic, and proteomic differences between PD hemispheres correspond to the lateralization of PD symptoms, with abnormalities being most prevalent in the hemisphere matched to side of symptom predominance. Hemispheric asymmetry and symptom lateralization in PD is linked to genes affecting neurodevelopment, immune activation, and synaptic transmission. PD patients with a long disease course have greater hemispheric asymmetry in neuronal epigenomes than those with a short disease course.

Conclusions
Hemispheric differences in epigenetic gene regulation are prevalent in neurons and may affect the progression and symptoms of PD.*

________________________________________

Comment 3: There are several places in the manuscript where the conclusions about DNA methylation differences detected are overstated. First, in the title, "Epigenetic contributions to" should be replaced with something more like "Epigenetic differences in". The last sentence of the Introduction reading "Together, our results support that hemispheric asymmetry in PD involves the disrupted epigenetic regulation of genes, which may enable asymmetric neuronal dysfunction and the contralateral predominance of PD symptoms" is not supported by the data and should be similarly reworded to remove the implication of epigenetic causality. Also, in the Discussion, statements such as "CpH silencing of gene activity" are not appropriate because neither this or prior studies of CH methylation have established its causality in gene repression.

*Reponse 3: The reviewer makes a good point and we have revised the title, and text in the Introduction and Discussion. The following revisions have been made:

Title, pg 1. Hemispheric asymmetry in the human brain and in Parkinson’s disease is linked to divergent epigenetic patterns in neurons

Introduction, pg 5. Together, our results support that neurons of PD patients have pronounced hemispheric differences in DNA methylation that are associated with the lateralization of PD symptoms.

Discussion, pg 17. Neuronal asymmetry in CpH at gene regulatory elements*

________________________________________

Comment 4: The Discussion did not really address the main findings of the manuscript and how they fit into the existing literature on the DNA methylation landscape of the brain in health and disease. CpH methylation in development and other disorders should be discussed, particularly. It is still unclear if CpH methylation in neurons is a cause or consequence of differential gene expression patterns.

*Response 4: We have added text to the discussion regarding the DNA methylation landscape in health and disease, particularly on CpH. However, we maintained a discussion focused on interpreting hemispheric asymmetry in neuronal DNA methylation. The following text has been added to the discussion:

Discussion, pg 17. Neurons exhibit a prominent gain in CpH methylation in the first five years of life, during a period of major synaptic restructuring [1,2,3]. Changes in CpH methylation status in early life have been implicated in synapse formation, neurotransmission, and alternative splicing [1,2]. Neuronal CpH methylation changes with aging have also been associated with neurodegenerative and neurocognitive disorders [1,4]. In our study of adult brain neurons, hemispheric asymmetry in CpH methylation preferentially affected genes involved in neurodevelopment, synaptic organization, and brain diseases, including neurodegenerative illnesses. Neuronal asymmetry in CpH methylation at gene regulatory elements may instill a differential vulnerability of hemispheres to synaptic dysfunction and neuronal loss that lead to neurodegenerative diseases. In support, hemispheric asymmetry in DNA methylation for PD patients was largely driven by changes in CpH methylation.

References
1: Price AJ, Collado-Torres L, Ivanov NA, Xia W, Burke EE, Shin JH, Tao R, Ma L, Jia Y, Hyde TM, Kleinman JE, Weinberger DR, Jaffe AE. Divergent neuronal DNA methylation patterns across human cortical development reveal critical periods and a unique role of CpH methylation. Genome Biol. 2019 Sep 26;20(1):196. doi: 10.1186/s13059-019-1805-1. PubMed PMID: 31554518; PubMed Central PMCID: PMC6761727.

2: Lister R, Mukamel EA, Nery JR, Urich M, Puddifoot CA, Johnson ND, Lucero J, Huang Y, Dwork AJ, Schultz MD, Yu M, Tonti-Filippini J, Heyn H, Hu S, Wu JC, Rao A, Esteller M, He C, Haghighi FG, Sejnowski TJ, Behrens MM, Ecker JR. Global epigenomic reconfiguration during mammalian brain development. Science. 2013 Aug 9;341(6146):1237905. doi: 10.1126/science.1237905. Epub 2013 Jul 4. PubMed PMID: 23828890; PubMed Central PMCID: PMC3785061.

3: Stroud H, Su SC, Hrvatin S, Greben AW, Renthal W, Boxer LD, Nagy MA, Hochbaum DR, Kinde B, Gabel HW, Greenberg ME. Early-Life Gene Expression in Neurons Modulates Lasting Epigenetic States. Cell. 2017 Nov 16;171(5):1151-1164.e16. doi: 10.1016/j.cell.2017.09.047. Epub 2017 Oct 19. PubMed PMID: 29056337; PubMed Central PMCID: PMC5693680.

4: Li P, Marshall L, Oh G, Jakubowski JL, Groot D, He Y, Wang T, Petronis A, Labrie V. Epigenetic dysregulation of enhancers in neurons is associated with Alzheimer's disease pathology and cognitive symptoms. Nat Commun. 2019 May 21;10(1):2246. doi: 10.1038/s41467-019-10101-7. PubMed PMID: 31113950; PubMed Central PMCID: PMC6529540.*

Reviewer #2

Comment 1: The authors show that the majority of the methylation differences are from CH methylation in normal brain samples. Did the author separate the individuals with left or right preference? I assume the methylation will correlate with this preference, but I can't figure out through the manuscript whether this has been corrected before the analysis.

*Response 1: In our brain cohorts we do not have information on whether the individual was left or right handed. However, ~90% of individuals have a right hand preference, as demonstrated in large populational studies [1-3]. For the PD patients, we had information of the body side of symptom dominance (which we investigated, see Figure 2-3), but did not have information on handedness. Though some studies of PD show an association between hand preference and hemispheric asymmetry in neurodegeneration [4-5], this effect is relatively modest and not replicated in all studies [6-7]. Hence, hemispheric asymmetry in PD is largely not explained by preferential body side usage. The following text has been added to the Discussion and Methods:

Discussion, pg 18: The factors rendering neurons more vulnerable in one brain hemisphere over the other is one of the most enigmatic puzzles of PD. Previously, it has been proposed that symptom lateralization in PD is related to body side preference (handedness), though this effect is relatively modest and not replicated in all studies [4-7]. Our epigenetic, transcriptomic, and proteomic analyses of hemispheric differences in PD, relative to controls, converges on gene pathways involved in neurodevelopment, immune activation, and synaptic transmission. Analysis of phenotypic pathways associated with hemispheric asymmetry in PD were strongly related motor dysfunction, further supporting the relevance of the genes identified in this study to PD. Specifically, we found that hemispheric asymmetry in PD was linked to the dysregulation of NCAM1. NCAM1 exerts a regulatory role on the development and survival of dopaminergic neurons [8], and also impacts immune responses [9]. Our analysis of genetic-epigenetic interactions indicates that NCAM1 abnormalities in PD may have a genetic basis, and consequently may precede disease onset. In addition, we found hemispheric differences in α-synuclein, which has a central role in Lewy pathology in PD [10]. In experimental models, selective vulnerability of brain regions to the spread of synucleinopathy and neuronal death was closely correlated with neuronal connectivity and endogenous α-synuclein levels [11,12]. Hence, molecular asymmetries, particularly those affecting neuronal development and survival, neurosignaling, and immune activation, may render one hemisphere more vulnerable to degenerative processes in PD.

Methods, pg 20. Information on handedness was not available for controls and PD patients.

References
1. de Kovel CGF, Carrión-Castillo A, Francks C. A large-scale population study of early life factors influencing left-handedness. Sci Rep. 2019 Jan 24;9(1):584. doi: 10.1038/s41598-018-37423-8. PubMed PMID: 30679750; PubMed Central PMCID:
PMC6345846.

2. Peters M, Reimers S, Manning JT. Hand preference for writing and associations with selected demographic and behavioral variables in 255,100 subjects: the BBC internet study. Brain Cogn. 2006 Nov;62(2):177-89. Epub 2006 Jun 23. PubMed PMID:16797814.

3. Gilbert AN, Wysocki CJ. Hand preference and age in the United States. Neuropsychologia. 1992 Jul;30(7):601-8. PubMed PMID: 1528408.

4: van der Hoorn A, Burger H, Leenders KL, de Jong BM. Handedness correlates with the dominant Parkinson side: a systematic review and meta-analysis. Mov Disord.2012 Feb;27(2):206-10. doi: 10.1002/mds.24007. Epub 2011 Oct 12. Review. PubMed PMID: 21994149.

5: Barrett MJ, Wylie SA, Harrison MB, Wooten GF. Handedness and motor symptom
asymmetry in Parkinson's disease. J Neurol Neurosurg Psychiatry. 2011Oct;82(10):1122-4. doi: 10.1136/jnnp.2010.209783. Epub 2010 Sep 22. PubMed PMID:20861062; PubMed Central PMCID: PMC3729350.

6: Stochl J, Hagtvet KA, Brozová H, Klempír J, Roth J, Růzicka E. Handedness does not predict side of onset of motor symptoms in Parkinson's disease. Mov Disord. 2009 Sep 15;24(12):1836-9. doi: 10.1002/mds.22653. PubMed PMID: 19514074.

7: van Rooden SM, Visser M, Verbaan D, Marinus J. Handedness associated to side of onset of Parkinson's disease? Parkinsonism Relat Disord. 2009 Aug;15(7):546-7. doi: 10.1016/j.parkreldis.2008.11.002. Epub 2008 Dec 13. PubMed PMID: 19073372.

8: Xiao MF, Xu JC, Tereshchenko Y, Novak D, Schachner M, Kleene R. Neural cell adhesion molecule modulates dopaminergic signaling and behavior by regulating dopamine D2 receptor internalization. J Neurosci. 2009 Nov 25;29(47):14752-63.doi: 10.1523/JNEUROSCI.4860-09.2009. PubMed PMID: 19940170; PubMed Central PMCID:PMC6666007.

9: Van Acker HH, Capsomidis A, Smits EL, Van Tendeloo VF. CD56 in the Immune System: More Than a Marker for Cytotoxicity? Front Immunol. 2017 Jul 24;8:892. doi: 10.3389/fimmu.2017.00892. eCollection 2017. Review. PubMed PMID: 28791027; PubMed Central PMCID: PMC5522883.

10: Shahmoradian SH, Lewis AJ, Genoud C, Hench J, Moors TE, Navarro PP, Castaño-Díez D, Schweighauser G, Graff-Meyer A, Goldie KN, Sütterlin R, Huisman E, Ingrassia A, Gier Y, Rozemuller AJM, Wang J, Paepe A, Erny J, Staempfli A, Hoernschemeyer J, Großerüschkamp F, Niedieker D, El-Mashtoly SF, Quadri M, Van IJcken WFJ, Bonifati V, Gerwert K, Bohrmann B, Frank S, Britschgi M, Stahlberg H, Van de Berg WDJ, Lauer ME. Lewy pathology in Parkinson's disease consists of crowded organelles and lipid membranes. Nat Neurosci. 2019 Jul;22(7):1099-1109.doi: 10.1038/s41593-019-0423-2. Epub 2019 Jun 24. PubMed PMID: 31235907.

11: Henderson MX, Cornblath EJ, Darwich A, Zhang B, Brown H, Gathagan RJ, Sandler RM, Bassett DS, Trojanowski JQ, Lee VMY. Spread of α-synuclein pathology through the brain connectome is modulated by selective vulnerability and predicted by network analysis. Nat Neurosci. 2019 Aug;22(8):1248-1257. doi: 10.1038/s41593-019-0457-5. Epub 2019 Jul 25. PubMed PMID: 31346295; PubMed Central PMCID: PMC6662627.

12: Zheng YQ, Zhang Y, Yau Y, Zeighami Y, Larcher K, Misic B, Dagher A. Local vulnerability and global connectivity jointly shape neurodegenerative disease propagation. PLoS Biol. 2019 Nov 21;17(11):e3000495. doi: 10.1371/journal.pbio.3000495. eCollection 2019 Nov. PubMed PMID: 31751329.*

________________________________________

Comment 2: When analysis these sites, they should separate the CpG and CH sites into three different groups: promoter, distal regulatory regions, and gene body. Depends on the genomic location, CpG methylation is correlated with various genomic activities.

*Response 2: In our study, hemispheric asymmetry in DNA methylation was profiled at enhancers and promoters. To further examine the genomic context of differentially methylated enhancers and promoters, we examined whether specific genomic locations had an enrichment in differentially methylated sites associated with hemispheric asymmetry in controls and in Parkinson’s disease patients. We did not observe an enrichment of a specific type of genomic location. This analysis is now displayed in Figure S4.

Figure S4. Genomic locations of differentially methylated neuronal enhancer/promoter cytosines associated with hemispheric asymmetry in controls and in PD patients. Location of enhancer/promoter cytosine sites relevant to hemispheric asymmetry in controls (a) and differential hemispheric asymmetry in PD patients relative to controls (b). The y-axis shows the percent number of cytosines with significant hemispheric asymmetry in DNA methylation (DMC), relative to background cytosines.
________________________________________

Comment 3: Figure 1c, where shows the genomic distances to TSS for differential methylated sites, the y-axis should be scaled at the same level between the left and right plot. The current plots are too zoomed out, therefore hard to tell the distance from TSS, which was only average at 47kb, while the intervals on the plots are at 250kb. To better display the data, I suggest the authors use a CDF plot to show the distribution of distance to TSS.

Response 3: In agreement with the reviewer’s request, we have revised Figure 1c to have the same y-axis scale for CpG and CpH. Since enhancers can be distally located from the TSS, we retained a zoomed-out plot, but have now added an inset with a zoomed-in view. The figure shows the number of differentially methylated cytosines upstream and downstream of the TSS. The revised Figure 1c is:

Fig. 1 (c) Genomic location of DNA methylation changes involved in hemispheric asymmetry in human cortical neurons. The number of CpG (left panel) and CpH (right panel) sites with increased DNA methylation in the left or right hemisphere is shown. Inset is a close-up of the transcription start site region.

Also we edited the relative text.
Results, pg 6. In neurons, the hemispheric asymmetry of DNA methylation most often affected cis-acting regulatory elements, located proximal to transcription start sites (peak within 5 kb and average distance 47.2 ± 1.4 kb from transcription start sites; average DNA methylation change 6.09 ± 0.39% at CpGs and 1.34 ± 0.05% at CpHs (Fig. 1c; Additional file 1: Figure S4 and S5).*
________________________________________

Comment 4: The authors used Schmitt et al., 2017 Cell Reports to link distal regions to target genes. However, HiC datasets were not sequenced deep enough to call interactions between gene and regulatory regions reliably. The authors should use Song et al., Nature Genetics 2019 in which they mapped promoter-centric interactions genome-wide in excitatory neurons and Jung et al., Nature Genetics 2019 with promoter centric interactions data in brain tissues. This analysis affects figure 1e, 2a, 2d, 2e,3c, and 3d.

*Response 4: We thank the reviewer for this suggestion to improve our chromatin conformation analysis. We have revised our chromatin interaction analysis and gene annotation throughout our study. We now use the Song et al., Nature Genetic 2019 interactions in excitatory neurons [1]. We also use the recent Nott et al., 2019 Science, which has promoter-centric interactions, genome-wide, in cortical neurons isolated by flow cytometry (NeuN+ nuclei, as described in our study) [2]. We did not use the Jung et al., Nature Genetics 2019 because it examined interactions in bulk brain tissue. We have revised the Figures and Methods accordingly.

References
1: Song M, Yang X, Ren X, Maliskova L, Li B, Jones IR, Wang C, Jacob F, Wu K,
Traglia M, Tam TW, Jamieson K, Lu SY, Ming GL, Li Y, Yao J, Weiss LA, Dixon JR,
Judge LM, Conklin BR, Song H, Gan L, Shen Y. Mapping cis-regulatory chromatin
contacts in neural cells links neuropsychiatric disorder risk variants to target
genes. Nat Genet. 2019 Aug;51(8):1252-1262. doi: 10.1038/s41588-019-0472-1. Epub
2019 Jul 31. PubMed PMID: 31367015; PubMed Central PMCID: PMC6677164.

2: Nott A, Holtman IR, Coufal NG, Schlachetzki JCM, Yu M, Hu R, Han CZ, Pena M,
Xiao J, Wu Y, Keulen Z, Pasillas MP, O'Connor C, Nickl CK, Schafer ST, Shen Z,
Rissman RA, Brewer JB, Gosselin D, Gonda DD, Levy ML, Rosenfeld MG, McVicker G,
Gage FH, Ren B, Glass CK. Brain cell type-specific enhancer-promoter interactome
maps and disease-risk association. Science. 2019 Nov 29;366(6469):1134-1139. doi:
10.1126/science.aay0793. Epub 2019 Nov 14. PubMed PMID: 31727856.*

________________________________________

Comment 5: Similarly, in Figure 2a, where the authors tried to show PD related genes show differential methylation, it was not clear whether this is based on TSS or distal region methylation (either CG or CH). How do these methylation differences affect gene expression? Is this PD gene more enriched than the background?

*Response 5: We appreciate the request for further clarification on the GWAS genes highlighted in Figure 2a. We have the following details in the Figure legend and Methods:

Figure legend, pg 45. Highlighted are genes implicated in PD risk (familial and/or identified by GWAS [48]) that have 3 or more methylated cytosines in enhancers or promoters significantly associated hemispheric asymmetry in PD.

Methods, pg 28. In the Manhattan plot comparing hemispheric asymmetry in PD patients relative to controls (Fig. 2a), PD risk genes identified in familial studies and/or by GWAS [50] are denoted. For figure clarity, we show the PD risk genes that have hemispheric differences in DNA methylation at least three cytosines in enhancers/promoters. Direction of DNA methylation asymmetry determined by the direction of the majority of significant cytosines, with ties resolved by the most significant cytosine.

DNA methylation and gene expression changes at PD GWAS genes are summarized in Figure 6. Among the genes with differentially methylated regulatory elements, we did not find an enrichment of PD GWAS genes, relative to background genes (p>0.05, hypergeometric test).*

________________________________________

Comment 6: I had a tough time understanding Figures 2d and 2e. I know the authors were trying to convey the idea of in PD samples specific methylation difference, but I suggest the authors make the figures more readable to the audience. Figure 1e has too many dimensions and doesn't need to be this complicated to show the same message. On the other hand, the corresponding text for explaining the results was too concise, offering little biological insights. This makes me feel they performed this analysis to fill in the figure without providing enough biological insight. This is when a lot of readers start to lose interest in the manuscript.

*Response 6: As suggested by the reviewer, we have added an image to help the readability of Figure 2d and reduced the complexity of Figure 2e. Only pathways with greater changes in the hemisphere matched to PD symptom dominance are shown. The revised Figure is:

Fig. 2. Epigenetic differences across hemispheres is increased in PD and is relevant to the lateralization of clinical symptoms. (a) Manhattan plot comparing the hemispheric asymmetry in DNA methylation in neurons of PD patients and controls, after adjusting for age, sex, postmortem interval, and neuronal subtype proportion. DNA methylation differences were profiled at enhancers and promoters in prefrontal cortex neurons of 57 PD patients and 48 controls. −log10(p) refers to the significance of differentially methylated cytosines (DMC), with the sign corresponding to the diagnosis group with greater hemispheric asymmetry. Threshold for genome-wide significance (red line) is q < 0.05. Highlighted are genes implicated in PD risk (familial and/or identified by GWAS [48][50]) that have 3 or more methylated cytosines in enhancers or promoters significantly associated hemispheric asymmetry in PDHemispheric differences in DNA methylation that affect genes linked to PD risk (familial and/or identified by GWAS [51]) are highlighted. (b) Comparison of the degree of hemispheric asymmetry in DNA methylation between PD patients and controls. The number of significant cytosine sites exhibiting more hemispheric asymmetry in PD or control neurons is shown. The p-value represents the enrichment of cytosines with greater hemispheric asymmetry in PD relative to controls, by Fisher's exact test. CpG and CpH contributions to hemispheric asymmetry are shown. (c) CpG and CpH involvement in hemispheric asymmetry changes in PD. The percent number of CpG or CpH sites significantly involved in hemispheric asymmetry changes in PD (relative to background) is shown. The enrichment of cytosine context was determined by hypergeometric test. (d) DNA methylation and transcriptional differences relevant to the lateralization of PD symptoms. Venn diagram showing the number of genes affected by differential methylation (left panel) or exhibiting differential expression (right panel) in the symptom-dominant (matched) or non-dominant (unmatched) PD hemisphere, relative to controls (DNA methylation: n = 17 PD-matched, 20 PD-unmatched, 48 controls; RNA-seq: n = 13 PD-matched, 11 PD-unmatched, 12 controls). (e) Pathway analysis of genes epigenetically and transcriptionally altered in neurons of the PD hemisphere matched or unmatched to side of symptom predominance, relative to control neurons. Only pathways clusters with nodes having greater changes in the hemisphere matched to PD symptom dominance are shown. Pathway enrichment analysis of DNA methylation data was done by g:Profiler (blue nodes) and of RNA-seq data was done by GSEA preranked (green nodes). Epigenetically and transcriptionally dysregulated pathways in PD hemispheres were merged in Enrichment Map and annotated by Autoannotate using q < 0.05 pathways. Dark green or dark blue nodes show greater disruption in symptom-dominant PD hemisphere; light green or light blue nodes show greater disruption in symptom non-dominant PD hemisphere (relative to controls). Pathways up- or down-regulated in PD hemispheres depicted by red or blue arrows, respectively, as determined by GSEA.*

________________________________________

Comment 7: I like the idea of replicating the study with an independent cohort. This is well done. But again, I would like to suggest changing the analysis of assigning target genes to distal regions with higher resolution HiC datasets. Also, consider an individual's preference of the left or right side in both control and PD samples when possible, as such information probably will help to provide cleaner analysis results.

*Response 7: Please see Reviewer 2, Response 1 and 4.*

________________________________________

Comment 8: The authors next perform proteomic analysis to identified differential proteins in PD samples. Is this protein level analysis correlated with the RNA-seq and methylome analysis?

*Response 8: The proteomics data examining hemispheric asymmetry in PD patients does not have a sample size that is sufficient for correlational analysis (n=3-5 individuals per group). However, we have now generated Additional file 10 summarizing the proteins exhibiting hemispheric asymmetry in PD that have corresponding gene transcripts and/or DNA methylation differences across PD hemispheres. Please see new Additional file 10.*

________________________________________

Comment 9: The section of "changes in hemispheric asymmetry in neurons with aging" is not well presented. Figure 4a needs labeling # of rows, and what are the different time points used for aging? Figure 4b, how are the values for each dot calculated? Is this a genome-wide average?

*Response 9: We have revised Figure 4a to have the labeling of number of rows and the different age time points. Figure 4a is also summarized in Figure 4b. In Figure 4b, each dot is an individual, with adjusted DNA methylation level on y-axis and individual’s age on the x-axis. Averaged adjusted DNA methylation values is shown for the sites significantly associated with aging (DNA methylation adjusted for sex, postmortem interval and neuronal subtype proportion). Data for the left (red) and right (blue) hemisphere is shown for controls (left panel) and Parkinson’s disease patients (right panel). The grey line represents confidence intervals. We have revised the Figure 4 and Figure 4 Legend as follows:

Fig. 4. Progressive loss of hemispheric asymmetry in DNA methylation with aging. Age-dependent changes in DNA methylation in neurons of the left and right hemisphere of PD patients and controls (n = 23 PD-left, 34 PD-right, 25 control-left, 23 control-right). (a) Heatmap showing adjusted DNA methylation levels at the 5,925 cytosine sites exhibiting significant changes in hemispheric asymmetry with aging (q < 0.05, robust linear model followed by contrasts; DNA methylation adjusted for sex, postmortem interval and neuronal subtype proportion). Cytosine sites with more aging changes in hemispheric asymmetry in PD (upper panels) or in controls (lower panels) are shown. The first and third quartiles of age for each group are shown in the top of the figure. (b) Scatter plot of aging changes in DNA methylation across hemispheres of controls and PD patients. Averaged adjusted DNA methylation values is shown for the sites significantly associated with aging (DNA methylation adjusted for sex, postmortem interval and neuronal subtype proportion). Data for the left (red) and right (blue) hemisphere is shown for controls (left panel) and PD patients (right panel). The grey area represents confidence intervals. (c) Concordance in aging changes in DNA methylation in the discovery and replication cohort. Venn diagram showing the significance of overlap between the discovery and replication cohorts in the genes with age-dependent changes in hemispheric asymmetry in DNA methylation. The p-value represents significance of overlap by hypergeometric test. (d) Top 10 genes involved in aging changes in hemispheric asymmetry. Genes affected by aging changes in DNA methylation were ranked using the discovery and replication DNA methylation datasets. Genes are listed by ranked order, and the heatmap shows adjusted rank significance score in each dataset.*

________________________________________

Comment 10: The last part of me-QTL analysis is interesting; besides some description, there is no figure or table associated with these results. What are the biological contributions of me-QTL to PD? What are the genes associated with me-QTL?

*Response 10: We thank the reviewer for suggesting to expand our reporting of the meQTL analysis. We now provide Additional file 11, which lists the genes relevant to hemispheric asymmetry in PD with genetic-epigenetic interactions, as determined in the meQTL analysis. We also performed a pathway analysis of the genes identified in the meQTL analysis. We find that genetic variation associated with inter-hemispheric differences in DNA methylation in PD is predominantly linked to genes affecting neurodevelopment. Also we found a strong pathway enrichment for NCAM1-mediated neurite outgrowth, synapse assembly and neuronal survival. Detailed pathway results are also listed in Additional file 11. Further, we found that SNP-associated DNA methylation changes across PD hemispheres were enriched at introns (p < 0.05, hypergeometric test). The results are now showing in Figure S10.

Figure S10. Pathways and genomic locations of DNA methylation changes relevant to hemispheric asymmetry in PD that were associated to genetic variation. meQTL analysis examining the effects of genotype on DNA methylation, adjusting for diagnosis, hemisphere, age, sex, postmortem interval, and neuronal subtypes (replication cohort, left and right hemispheres of n = 31 controls and 26 PD patients). Pathway maps (a) and biological processes (b) of SNP-associated DNA methylation sites relevant to hemispheric asymmetry in PD. Pathway analysis performed using MetaCore and q < 0.05 pathways are shown. (c) Genomic location of DNA methylation sites with inter-hemispheric differences in PD that are associated with genetic variation. Y-axis shows the percent number of SNP-associated DNA methylation sites relevant to hemispheric asymmetry in PD, relative to background cytosines. *p < 0.05, hypergeometric test.

Results, pg 16. In total, we found genetic variation to be associated with 211 genes that had inter-hemispheric differences in DNA methylation in PD (Additional file 11). Interestingly, pathway analysis revealed that these genes affect neurodevelopment and NCAM1-mediated neurite outgrowth, synapse assembly, and neuronal survival (Additional file 1: Figure S10; Additional file 11).*

**Second round of review**

**Reviewer 1**

Overall, the authors responded to the prior critiques appropriately and the manuscript is significantly improved.
However, there are still places where the causality of CH methylation was overstated and should be reworded:
1. p. 6 lines 42-45, "Indeed, CpH methylation, a silencing epigenetic mark at enhancers and promoters..." should be more like "CpH methylation, an epigenetic mark correlating with repression of enhancers and promoters in neurons"
2.  p. 17, lines 33-35, "At enhancers, CpH methylation acts as an epigenetic silencer and is highly associated with gene transcript levels" is not an accurate description of the references cited. A sentence more like "CpH methylation at enhancers is correlated with lower transcript levels..." would be more appropriate.
3. Abstract, Conclusions sentence: "epigenetic gene regulation" should be replaced by the more appropriate "DNA methylation patterns"

**Reviewer 2**

The authors improved the manuscript during revision and have addressed most of my concerns and expanded the analysis accordingly.

I have the following two suggestions to improve the manuscript.

1. In response to my previous comment 2, the authors break the CpG and CH  into several genomic locations. But what I meant to ask is whether they can observe any differentiation enrichment of methylation for both CpG and CH between promoters and enhancers. They should analysis CpG and CH groups separately.

2. Page 7, Line 54: "We then identified the gene targets of the enhancers and promoters, using the chromatin conformation data from human cortical neurons and excitatory neurons [47, 48], along with the in silico cis-regulatory element prediction tool, as described above. We found that PD patients had 4,691 genes with abnormal hemispheric asymmetry in epigenetic regulation, relative to controls (Additional file 6). " It is not clear whether the author used HiC data or GREAT or both to determine the target genes. Since GREAT is a distance-based tool for identifying target genes for enhancers, the authors shouldn't use it anymore unless no high-resolution HiC data is available in the corresponding cell types.

**Authors Response**

**Point-by-point responses to the reviewers’ comments:**

Reviewer #1: Overall, the authors responded to the prior critiques appropriately and the manuscript is significantly improved.

However, there are still places where the causality of CH methylation was overstated and should be reworded:

Comment 1: p. 6 lines 42-45, "Indeed, CpH methylation, a silencing epigenetic mark at enhancers and promoters..." should be more like "CpH methylation, an epigenetic mark correlating with repression of enhancers and promoters in neurons"

*Response 1: We have revised the text according to the reviewer's comment.*

*Results. Pg 6. Indeed, CpH methylation, an epigenetic mark correlating with repression of enhancers and promoters in neurons [21], was the primary epigenetic contributor to hemispheric asymmetry (2,371 CpHs and 216 CpGs, p < 10-34 for CpH enrichment, hypergeometric test; Fig. 1d).*

Comment 2: p. 17, lines 33-35, "At enhancers, CpH methylation acts as an epigenetic silencer and is highly associated with gene transcript levels" is not an accurate description of the references cited. A sentence more like "CpH methylation at enhancers is correlated with lower transcript levels..." would be more appropriate.

*Response 2: We have revised the text according to the reviewer's comment.*

*Discussion. Pg 17. At enhancers, CpH methylation is correlated with reduced gene transcript levels [21, 23].*

Comment 3: Abstract, Conclusions sentence: "epigenetic gene regulation" should be replaced by the more appropriate "DNA methylation patterns"

*Response 3: We have revised the text according to the reviewer's comment.*

*Abstract. Pg 2. Hemispheric differences in DNA methylation patterns are prevalent in neurons and may affect the progression and symptoms of PD.*

Reviewer #2: The authors improved the manuscript during revision and have addressed most of my concerns and expanded the analysis accordingly.

I have the following two suggestions to improve the manuscript.

Comment 1: In response to my previous comment 2, the authors break the CpG and CH into several genomic locations. But what I meant to ask is whether they can observe any differentiation enrichment of methylation for both CpG and CH between promoters and enhancers. They should analysis CpG and CH groups separately.

*Response 1: We calculated the enrichment in promoters and enhancers in CpG and CpH sites exhibiting hemispheric asymmetry in DNA methylation. We examined CpG and CpH sites separately. There was a* *significant enrichment in asymmetry-related changes in CpH methylation at enhancers in controls (p<10-22, hypergeometric test). We also observed an enrichment in hemispheric asymmetry in CpH methylation at promoters and enhancers in PD patients, relative to controls (p<0.01 and p<10-12, respectively, hypergeometric test). Notably, since promoters can at times act as enhancers [1], we clarified that this analysis examines CpG and CpH sites at enhancers and at gene transcription start sites (± 2 kb). This new analysis has been added to Figure S4c and S4d.*

*Figure S4. Genomic locations of differentially methylated neuronal enhancer/promoter cytosines associated with hemispheric asymmetry in controls and in PD patients. Location of enhancer/promoter cytosine sites relevant to hemispheric asymmetry in controls (a, c) and differential hemispheric asymmetry in PD patients relative to controls (b, d). (a, b) Genomic locations of enhancer/promoter cytosine sites with DNA methylation differences between hemispheres (relative to background of each location). (c, d) Distribution within enhancers and promoters of CpG and CpH sites exhibiting hemispheric asymmetry in DNA methylation. Enrichment of enhancers or transcription start site locations (TSS ± 2 kb) with CpG or CpH sites with differential methylation across hemispheres (CpG and CpH examined separately and relative to background). The y-axis shows the percent number of cytosines with significant hemispheric asymmetry in DNA methylation (DMC), relative to background cytosines. Enrichment determined by hypergeometric test.*

Comment 2: Page 7, Line 54: "We then identified the gene targets of the enhancers and promoters, using the chromatin conformation data from human cortical neurons and excitatory neurons [47, 48], along with the in silico cis-regulatory element prediction tool, as described above. We found that PD patients had 4,691 genes with abnormal hemispheric asymmetry in epigenetic regulation, relative to controls (Additional file 6). " It is not clear whether the author used HiC data or GREAT or both to determine the target genes. Since GREAT is a distance-based tool for identifying target genes for enhancers, the authors shouldn't use it anymore unless no high-resolution HiC data is available in the corresponding cell types.

*Response 2: To identify the gene targets of cis-regulatory elements investigated in this study, we used both Hi-C data from brain neurons and an in silico tool called GREAT. We have revised the text to clarify our use of this approach. We agree with the reviewer that if high-resolution Hi-C data is available in the exact cell types and sample demographics used in the study, then there is no need for additional tools to determine target genes. However, the two HiC neuronal datasets that currently exist are from human cortical neurons from the frontal and temporal cortex (ages 4-14) [2] and iPSC-induced excitatory neurons [3, 4]. Our bisulfite padlock probe sequencing was done on neurons isolated from the human prefrontal cortex of older adults. Since chromatin conformation can change with age, brain location, sex, and other factors [5-7], we find it necessary to add the in silico GREAT tool to ensure that we have effectively captured the interactions and gene targets.*

*Results. Pg.7-8. We then identified the gene targets of the enhancers and promoters, using the chromatin conformation data from human cortical neurons and excitatory neurons [47, 48] in combination with the in silico cis-regulatory element prediction tool, as described above. We found that PD patients had 4,691 genes with abnormal hemispheric asymmetry in epigenetic regulation, relative to controls (Additional file 6).*

*References*

*1.	Dao LTM, Galindo-Albarran AO, Castro-Mondragon JA, Andrieu-Soler C, Medina-Rivera A, Souaid C, Charbonnier G, Griffon A, Vanhille L, Stephen T, et al: Genome-wide characterization of mammalian promoters with distal enhancer functions. Nat Genet 2017, 49:1073-1081.*

*2.	Nott A, Holtman IR, Coufal NG, Schlachetzki JCM, Yu M, Hu R, Han CZ, Pena M, Xiao J, Wu Y, et al: Brain cell type-specific enhancer-promoter interactome maps and disease-risk association. Science 2019, 366:1134-1139.*

*3.	Song M, Yang X, Ren X, Maliskova L, Li B, Jones IR, Wang C, Jacob F, Wu K, Traglia M, et al: Mapping cis-regulatory chromatin contacts in neural cells links neuropsychiatric disorder risk variants to target genes. Nat Genet 2019, 51:1252-1262.*

*4.	Handel AE, Chintawar S, Lalic T, Whiteley E, Vowles J, Giustacchini A, Argoud K, Sopp P, Nakanishi M, Bowden R, et al: Assessing similarity to primary tissue and cortical layer identity in induced pluripotent stem cell-derived cortical neurons through single-cell transcriptomics. Hum Mol Genet 2016, 25:989-1000.*

*5.	Dekker J, Belmont AS, Guttman M, Leshyk VO, Lis JT, Lomvardas S, Mirny LA, O'Shea CC, Park PJ, Ren B, et al: The 4D nucleome project. Nature 2017, 549:219-226.*

*6.	Jung I, Schmitt A, Diao Y, Lee AJ, Liu T, Yang D, Tan C, Eom J, Chan M, Chee S, et al: A compendium of promoter-centered long-range chromatin interactions in the human genome. Nat Genet 2019, 51:1442-1449.*

*7.	Koohy H, Bolland DJ, Matheson LS, Schoenfelder S, Stellato C, Dimond A, Varnai C, Chovanec P, Chessa T, Denizot J, et al: Genome organization and chromatin analysis identify transcriptional downregulation of insulin-like growth factor signaling as a hallmark of aging in developing B cells. Genome Biol 2018, 19:126.*
